# Editorial: A molecular and structural approach to deciphering and combating infectious pathogens

**DOI:** 10.3389/fmicb.2026.1900349

**Published:** 2026-06-16

**Authors:** Valeria Napolitano, Eliana De Gregorio, Felipe Romero-Saavedra, Rita Berisio

**Affiliations:** 1 Institute of Biostructures and Bioimaging, Italian Research Council (CNR), Naples, Italy; 2 Department of Molecular Medicine and Medical Biotechnology, University of Naples Federico II, Naples, Italy; 3 Division of Paediatric Infectious Disease, Hauner Children's Hospital Ludwig-Maximilians-Universität München (LMU), LMU, Munich, Germany

**Keywords:** antimicrobial resistance (AMR), anti-virulence, infection, pathogen, protein structure

Microbial infections remain among the most persistent global health threats, causing millions of deaths each year and imposing a growing burden on healthcare systems worldwide. Bacteria, viruses, fungi, and parasites exploit sophisticated molecular strategies to invade host tissues, evade immune defenses and establish persistent infections. This challenge is further compounded by the rapid rise of antimicrobial resistance (AMR) and the emergence of novel pathogens, which together erode the efficacy of existing therapeutics. As conventional antibiotic pipelines stagnate, a new generation of studies is redefining how we understand, target and monitor microbial pathogenicity. The manuscripts in this Research Topic collectively illustrate a decisive shift: from empirical antimicrobial discovery toward a mechanistically informed, evolution-aware framework that integrates anti-virulence strategies, bio-derived multi-target agents, computational design, predictive analytics and vesicle-mediated biology.

A first conceptual pivot emerges from work on *Stenotrophomonas maltophilia*, a multidrug-resistant opportunistic pathogen. Stabile et al. demonstrate that the iminosugar L-NPDNJ, despite weak bactericidal activity, exerts additive effects when used in combination with amikacin and tobramycin, reducing their minimum inhibitory concentrations by two- to three-fold. At sub-inhibitory concentrations, the compound significantly modulates the expression of genes involved in antibiotic resistance, biofilm regulation, and virulence, including those encoding efflux pumps, global regulators, and extracellular proteases. These transcriptional changes translate into reduced virulence *in vivo* and enhanced macrophage clearance. This study underscores a critical insight: antimicrobial efficacy need not rely solely on killing. Disarming pathogens, rather than eradicating them, can attenuate disease severity while reducing selective pressure for resistance. Anti-virulence agents, particularly those that modulate host-pathogen interactions, represent a promising complement to traditional antibiotics.

A second thematic cluster of this Research Topic highlights the expanding potential of bio-derived, multi-target antimicrobials. Tiricz et al. dissect the antimicrobial region of NCR147, a rare non-cationic nodule-specific cysteine-rich peptide from *Medicago truncatula*. Through rational truncation and amino-acid substitution, they generate derivatives with enhanced potency, broad-spectrum activity and antibiofilm effects, including against *Acinetobacter baumannii*. These peptides act through a multi-hit mechanism involving membrane perturbation, efflux pump inhibition, and interference with intracellular targets. Importantly, fluorinated variants retain activity without cytotoxicity to human cells, illustrating how natural scaffolds can be engineered for therapeutic use. Complementing this, de Pascale et al. apply bio-orthogonal non-canonical amino-acid tagging (BONCAT) to track the inhibition of protein synthesis by proline-rich antimicrobial peptides (PrAMPs) in living *Escherichia coli* and *Klebsiella pneumoniae* bacteria. BONCAT, combined with flow cytometry, discriminates membrane-active peptides from those targeting intracellular processes and reveals rapid, long-lasting inhibition of translation. This methodological advance provides a powerful tool for mechanistic characterization of antimicrobial peptides, enabling precise mapping of their intracellular effects. Plant-derived antimicrobials extend beyond peptides. Ali et al. evaluate a peppermint oil nanoemulsion (PEONE) against multidrug-resistant *K. pneumoniae* and *Pseudomonas aeruginosa*. PEONE disrupts bacterial membranes, induces leakage of intracellular contents and exhibits sustained bactericidal activity. Molecular docking identifies caryophyllene as a key component with high affinity for β-lactamase enzymes, suggesting a dual mechanism of membrane disruption and enzyme inhibition. This work reinforces the therapeutic potential of natural products when combined with nanoscale formulation and computational analysis. Together, these studies signal a broader transition: natural molecules are no longer viewed as static entities but as rationally optimisable, multi-target antimicrobial platforms. Their capacity to act on membranes, intracellular pathways and resistance determinants simultaneously may reduce the likelihood of resistance emergence.

A third conceptual advance arises from computational acceleration of antimicrobial discovery. Qingxin et al. develop a multi-template homology modeling and virtual-screening pipeline to identify CNP0387675, a non-nucleoside inhibitor of MraY, an essential enzyme in peptidoglycan synthesis. Extensive molecular-dynamics simulations confirm stable binding to conserved catalytic residues. By circumventing the limitations of nucleoside analogs, this scaffold offers a promising approach to targeting Gram-negative pathogens. Such computational pipelines enable rapid exploration of chemical space, even in the absence of experimentally resolved structures, and can prioritize candidates for experimental validation. In parallel, Sedaghat et al. introduce HoloMoA, a holography- and deep-learning-based platform for mechanism-of-action (MoA) classification. Digital inline holographic microscopy captures time-resolved, label-free morphological responses of bacteria to antimicrobial exposure, while convolutional recurrent neural networks achieve 95% accuracy in MoA prediction across five functional classes. A Siamese neural-network architecture further enables novelty detection, distinguishing unseen mechanisms from known classes. This approach provides a scalable, affordable and unbiased tool for early-stage antimicrobial profiling, bridging the gap between computational discovery and mechanistic validation. Mechanistic insight must also be paired with surveillance of resistance evolution. Zhang et al. identify 53 mutations associated with decreased susceptibility to ceftriaxone in *Neisseria gonorrhoeae*, including 33 previously unreported variants. By integrating these mutations into a minimum-spanning-tree framework, the authors achieve improved sensitivity and specificity in predicting resistance compared with classical sequence typing.

A final thematic axis focuses on extracellular vesicles (EVs) and outer membrane vesicles (OMVs), which are now widely recognized as key mediators of bacterial communication, virulence and resistance. Ran et al. show that sub-inhibitory oxacillin exposure enhances EV secretion in oxacillin-sensitive MRSA and enriches vesicles with virulence-associated proteins, including penicillin-binding protein 2 and hemolysins. These EVs exhibit increased hemolytic activity, promote biofilm formation, stimulate pro-inflammatory cytokines and penetrate epithelial cells more effectively. Lu et al. provide a broader conceptual framework, reviewing the roles of OMVs in horizontal gene transfer, biofilm formation, immune modulation and antibiotic resistance. OMVs also hold promise as therapeutic platforms for vaccines, drug delivery and immune modulation. Together, these studies highlight vesicle biology as both a challenge and an opportunity: antibiotics can reshape vesicle-mediated communication, but vesicles themselves may be harnessed for anti-infective strategies.

Across these manuscripts, a coherent picture emerges. Antimicrobial innovation is shifting toward a mechanism-driven, evolution-aware paradigm that integrates anti-virulence approaches, multi-target natural products, computational design, and vesicle biology. This convergence offers a roadmap for revitalizing antimicrobial pipelines by prioritizing agents that disarm rather than solely kill, engineering natural scaffolds for multi-hit activity, exploring novel chemical space, and incorporate vesicle-mediated processes into both therapeutic design and stewardship policies. The future of antimicrobial therapy will depend on embracing this multidimensional strategy. By aligning mechanistic insight with technological innovation, the field can move beyond incremental modifications of existing antibiotics toward transformative solutions capable of outpacing microbial evolution.

